# “The war phone”: mobile communication on the frontline in Eastern Ukraine

**DOI:** 10.1057/s42984-022-00049-2

**Published:** 2022-10-21

**Authors:** Roman Horbyk

**Affiliations:** Södertörn University, Stockholm, Sweden

**Keywords:** Mediatization, War, Mobile phone, Mobile communication, Ukraine, Donbas

## Abstract

One of the problems in the growing subfield of mediatization of war is evidence on how exactly civilian communication devices become integrated with warfare. In this article, I focus on patterns of use of mobile phones on the frontline in Eastern Ukraine. Based on qualitative in-depth interviews with Ukrainian servicemen and women, this article presents a typology for the frontline use of mobiles in the spirit of actor–network theory. The omnipresence of mobiles on the battlefield creates a set of unique participatory media practices. A variety of personal purposes, such as private communication and entertainment, are combined in the same device with wiretapping, fire targeting, minefield mapping and combat communication. Mobiles supplant old or unavailable equipment and fill gaps in military infrastructure, becoming weaponized and contributing to the hybridization of the military and the intimate, and of war and peace. These results imply the role of mobiles as a mediated extension of battlefield and question the very definition of what constitutes weapon as tool of combat.

## Introduction

Mobile phone has become like a toothbrush: an ordinary, indispensable object that almost everybody has. What happens when civilians become soldiers and arrive on the battlefield, everyone with a mobile in their pockets? How does that change the battlefield itself?

In this article, I focus on the role of mobile phone on the East Ukrainian frontline. There is already growing yet unsystematic evidence of the broad and unorthodox use of mobile communication and its infrastructure in the ongoing Russo-Ukrainian War, mostly in its 2014–2021 phase (I refer to this phase as the War in Donbas). In the spirit of actor–network theory (Latour 2005), I trace the difficult frontline history of the mobile phone, where, despite being formally banned, it became indispensable for the military as its informal communication system, combat tool and entertainment station. The study highlights, primarily by way of 16 in-depth interviews with Ukrainian soldiers who were deployed on the frontline in 2014–2019 as well as some of their family members, how connectivity, mediatization and war forged a specific “media amalgamation” (Jansson 2014, 2018), a particular way of associating certain media with a certain location (such as the frontline), and created a set of affordances, or action possibilities (Norman 1999), that come at a certain price. Not only they allow the soldier to take their home to the frontline but simultaneously bring war to the soldier’s home as well.

This article is structured as follows. First, it departs from the gaps identified in previous research and description of the theoretical framework that highlights both the role of mobile phones as a non-human actor in war (Latour 2005) and the participation culture of their users (Jenkins 2006; Carpentier 2016). Furthermore, I provide a concise description of methodology and background to the War in Donbas as a phase in the Russo-Ukrainian War in the following sections. After this, the empirical findings are presented, focusing on the technical infrastructure, media ecology/texture of the frontline, informal rules around use of particular communication media/channels, technological improvisation, personal and combat communication at the frontline, other combat-specific uses (wiretapping, targeting/spotting, fire control, mapping), as well as entertainment. The findings, summarized in the conclusion, indicate that, while certain practices are differentiated between specialized channels, mobile phone integrates private uses with combat-specific uses in the same device, which leads to a number of hybridizations and blurred boundaries between private/public, civilian/military and intimate/official.

## From information war to war information: on gains and gaps in previous research

The uses of communication around the conflict, such as fake news and propaganda, have already garnered attention in academic literature (e.g., Horbyk 2015; Khaldarova and Pantti 2016; Bolin et al. 2016). Yet there is virtually no inquiry into how actual combatants communicate on the ground. Käihkö (2018) noted research implications of mobiles’ omnipresence on the frontline in his reflection on own experiences of “chatnography”, a messenger-mediated ethnography of Ukrainian volunteer battalions. However, the use of mobiles in combat and life on deployment fell completely outside his scope.

Shklovski and Wulff (2018) collected undoubtedly interesting material through a study design that also opens it for criticism. Lumping together civilians and combatants on both sides, also around Donbas and in Crimea, is counterproductive because of the structural incomparability of their experiences. Civilians in Western Russia, unlike those in Eastern Ukraine, did not experience occupation, warfare or shelling in 2014–2021. The conflation of experiences at a protest (such as Euromaidan) and in an all-out war (such as in Donbas) is similarly misleading, just as it is hard to put on the same comparative level the disorganized Ukrainian Armed Forces (UAF) of 2014 and the fully hybrid invasion force with nuclear weapons behind (cf. Freedman 2014). The authors are also oblivious of the situation’s development in time. Some of the uses of mobiles they indicated—communication and organizing, fire targeting and surveillance—are also confirmed in this study. However, Shklovski and Wulff’s broad focus let many important aspects slip under radar, which the present study also demonstrates. Boichak (2021) as well as Boichak and Jackson (2019) highlighted how Ukrainian civilian activists in the warzone used technological affordances to mobilize themselves as a networked public and rearticulate their national identity. Yet, how does that look like for the military?

Beyond these early approaches to the Ukrainian case, similar research in other contexts highlighted the key role of mobile communication in warfare. However, even here evidence is scarce. In Merrin (2018), an entire chapter is dedicated to wearables, mobiles and augmented reality in war without almost any references to empirical studies on mobiles in war. Scholars mostly focused on smartphone use by US and Israeli soldiers (Lawson 2014; Rosenberg 2018; Gardner 2019), typically in quieter and more routine environments. A common finding is that the use of social media and mobiles by soldiers in peace, as Maltby and Thornham (2016) found, reflects civilian “digital mundane”. However, these studies mostly focus on mobile phone uses on the bases or in peacetime and rarely pinpoint how mobile communication transforms during an actual *combat* situation—and transforms it. Moreover, there has been no attempt to link such transformations with the unique role that mobile phone plays in conflict as a non-human actor and how this role is exploited through participatory practices. This paper aims to fill exactly this gap with empirical material from an actual frontline, with a focus on the mobile as actor in the sense of “…any thing that does modify a state of affairs by making a difference” (Latour 2005, 71).

## Media textures of war: theoretical premises

Indeed, it is actor–network theory that is promising because of its focus on the complexity of roles technology can play during formation of human associations. Latour’s suggestion is to consider the means to form them not as intermediaries, or passive enactors of a required function, but as mediators that are complex, composed of other mediators, and can act unpredictably. Technologies and devices are thus considered actors (though of course not agents with an own will) on par with humans, leading to differentiation of human and non-human actors that together form complex networks. These networks can act as a higher-level actor while remaining composite and internally complex. Tracing the intricacies of these networks, their figurations and concatenations of mediators that produce certain effects only in combination is the task of actor–network theory (ibid., 62), one that is suited to untangling the warzone’s crowded communication webs. This particularly means attending to how “things might authorize, allow, afford, encourage, permit, suggest, influence, block, render possible, forbid, and so on” (ibid., 72).

This perspective allows bringing mobile phone and its role—how exactly it makes a difference!—to the fore. At the same time, it must be balanced with a perspective that does justice to the human users’ agency. Jenkins (2006) proposed the concept of participatory culture, under which any individual regardless of their status or skills may consume and produce at once, thus creating new content and new ideas. Social media and mobile communication are often considered within the framework of participatory culture and produsage, whereby users are simultaneously content producers who collaborate on creating and improving the content (Bruns 2008; Bruns and Schmidt 2011).

Nico Carpentier pointed out two possible understandings of participation:

In [the sociological] approach, participation includes many (if not all) types of human interaction, in combination with interactions with texts and technologies. Power is not excluded from this approach, but remains one of the many secondary concepts to support it. […] Participation simply describes how users in one way or another contribute to or participate in using a service or a platform. […] In contrast, the political approach produces a much more restrictive definition of participation, which refers to the equalisation of power inequalities in particular decision-making processes (see Carpentier 2011; Carpentier, Dahlgren, and Pasquali 2014), (Carpentier 2016, 71–72).

The study of “participative war” has so far mainly concentrated on the (self)mobilization of civilians (cf. Merrin 2018, 195–217; Boichak 2021), yet not so much has been written on how soldiers themselves engage in participatory communication practices, apart from milblogs. In this article, I am highlighting not only how participation functions in exceptional circumstances at the frontline but how it is stimulated by such extreme environment. Furthermore, the findings indicate whether the regulated and hierarchical power regime of the military in war still leaves some space to read soldiers’ participation from the second, political perspective as outlined by Carpentier.

How people have communicated at a frontline in the 2010s and 2020s is impossible to ponder without the background of overall integration of media and communication technologies with all social spheres. This integration is captured by the concept of mediatization, or even “deep mediatization”, “an advanced stage of the process in which all elements of our social world are intricately related to digital media and their underlying infrastructures” (Hepp 2020, 5). Its inherent feature is the provisional, evolving and unfixed “culture of connectivity” (van Dijk 2013, 20–21). Based on the human need and connectedness, the automated connectivity makes sociality coded and affected by algorithms, the profit-driven and commoditizing logics, and erasure of boundaries between the public, the private and the corporate.

Mediatization and connectivity permeate even exceptional and extreme situations. Contemporary warfare is particularly characterized by hyperconnectivity and omnipresence of portable communication devices, which makes it “digital” (Merrin 2018)—or, as provocatively suggested by O’Loughlin, a “post-digital war”, whereby new technologies “have already been integrated into how militaries, media and societies wage, resist and understand war” (O’Loughlin 2020, 123). The early consequences of mediatized warfare were theorized as the diffused war, characterized by chaotic and unpredictable communication flows, which, however, did not last long as the military fully harnessed the potential of new media and learned to control and influence that initial chaos—this latest stage of war mediatization is defined as “arrested war” (Hoskins and O’Loughlin 2015).

Since frontline is an extraordinary *place*, it is also necessary to consider its mediatization as a socio-*spatial* phenomenon. In this approach, media become indispensable for organizing space materially, by giving it a “texture” (Jansson 2014; 2018) and creating “media amalgamations”, in which different media are entangled with different places or practices. This perspective also problematizes autonomy: while media enable certain actions, they also create dependence on themselves (Jansson 2018, 7–9). Media connectivity is perceived as both an asset and a liability with “a sense of simultaneously gaining and losing control” (Jansson 2018, 16). This theoretical focus highlights how mobile phones and infrastructures that support them become indispensable, how they premediate certain actions and how these are normalized as social practices (Jansson 2014, 278–279), discerning the internal contradictions of this change and how they play out in people’s adapting and resisting to them.

Frontline magnifies these effects manifold, as every combination of risks and advantages at any given location with its particular media amalgamation becomes a matter of life and death. This magnified situation is best understood conceptually in terms of distinct media ecologies (or textures in Jansson’s terminology) because it emphasizes media as an environment in its own right, complex and evolving, and urges to consider mobiles in the context of niches occupied, on the one hand, by military field phones, radios and satellite devices, and, on the other hand, by civilian tablets and laptops.

## The War in Donbas as a case study

The material in this study comes from the War in Donbas, and the results are valid for this conflict. I consider the War in Donbas a part of the larger Russo-Ukrainian War, ongoing since 2014. This larger war started with the annexation of Crimea by Russia (Käihkö 2021) and includes the continuing 2022 full-scale invasion of Ukraine by Russia. The beginning of the Donbas War as a phase in this greater conflict can be dated to 12 April 2014 when a group of Russian paramilitaries led by an FSB operative Igor Girkin (Strelkov) took over the city of Sloviansk, leading to first armed clashes in the region (Portnov 2016). The conflict quickly grew in scope, and the Ukrainian Armed Forces, after reclaiming some ground during the summer of 2014, were defeated by Russian conventional military units at the Battle of Ilovaisk (Käihkö 2021). The conflict stabilized somewhat through first Minsk agreements before a further Russian offensive claimed Debaltseve in February 2015, followed by the second Minsk accords (Wittke 2019). This created a more or less stable frontline with only minor changes until 2022 (Käihkö 2021). The war since 2015 was mostly a positional, static conflict; while it is sometimes characterized as a small or “limited war” (Freedman 2014), it had a significant scope most of the time, involving thousands of combatants and organized militaries on both sides (the Russian proxy force being run and to a large extent manned by Russian officers and former servicemen; see Hosaka 2019). The use of volunteer units decreased after the initial period, and the conflict became professionalized and routinized to some degree. I adopt here the definition of the Donbas War as an example of hybrid warfare (cf. Hoffman 2007; Fleming 2011). I am aware of the conceptual debates around the term but they fall out of scope of my contribution; “hybrid war” is applied here as a convenience label to contrast the War in Donbas with the 2022 full-scale conventional Russian invasion. Hybrid war also captures such aspects of 2014–2021 as the complexity of adversary (combining state and non-state actors), a special focus on disruption and subversion, limited use of certain types of weapons and military branches (such as aviation), an especially significant role carried by information warfare, disinformation, public diplomacy and other weaponized non-kinetic instruments, but above all conversion of non-military means to serve military purposes, which is particularly expressed in the combat uses of mobile phone.

## The note on methods, ethics and sample

To tell the story of the mobile phone at this Donbas frontline between 2014 and 2022, I had to rely on human witnesses—the phones may be often muted but are always mute. From 2019, I conducted in-depth unstructured (given a diverse sample and a sensitive subject) and semi-structured interviews with soldiers who were deployed at the frontline in Eastern Ukraine and participated in combat anytime during the ongoing conflict. The earliest deployment in the sample dates to spring of 2014 and the latest to spring of 2019. (The frontline media amalgamation arguably changed little between 2019 and early 2022.) The study deals with potentially vulnerable participants, but the conversations’ content did not concern sensitive information per se, although references to sensitive issues (such as violations of rules) could occur. To keep research ethical, I ensured the following: 1) clear explanation of the purpose, methods and outcomes of the study to the participants; 2) procurement of explicit consent from all the participants; 3) ensuring the participants’ confidentiality. In particular, I refrained from assigning even fictional names to them; where necessary or significant, I simply refer to their rank or type of troop, or place of origin. I consciously chose not to make this profiling consistent to complicate the identification of soldiers.

**Fig. 1:**
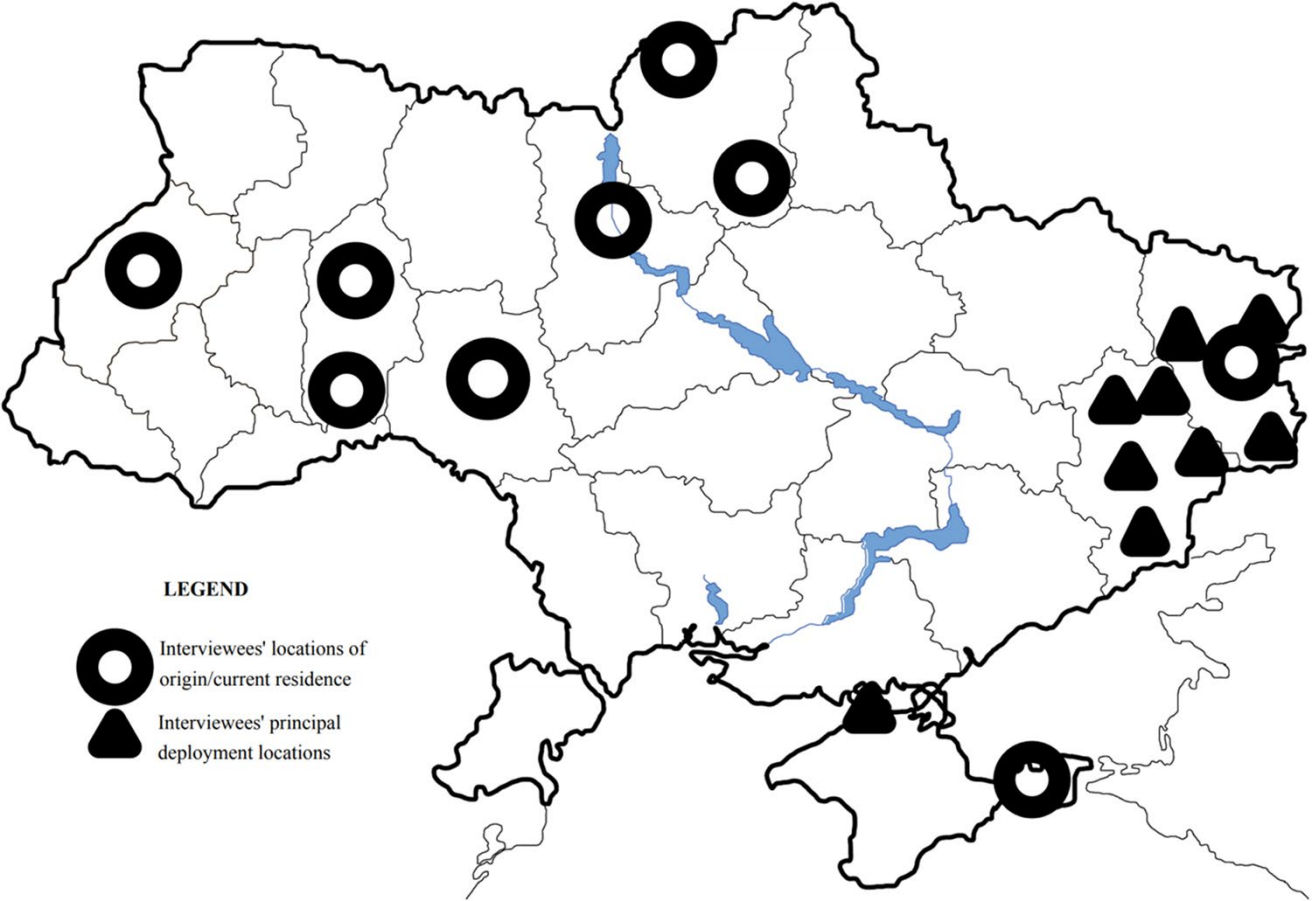
Territorial distribution of the interviewees’ places of origin and deployment

This article is based on 16 in-depth one-on-one interviews with 14 members of the military and two spouses. Some of the interviews were carried out face-to-face while other in distance mode using Zoom during covid-19 restrictions, which is estimated as an acceptable alternative with little impact on the data quality (Gray et al. 2020). I tried to diversify the pool of participants as much as possible, both in terms of origin and social profile. The regional provenance of participants as well as the location where they acquired experiences discussed during interviews are summarized in Fig. 1. Overall, the composition is diverse and includes men and women, privates and officers up to the rank of major, members of the UAF and volunteer battalions, soldiers in the infantry, an armoured vehicle driver, a scout, a pioneer, an artillerist, paramedics, signal troops, a sniper and one chaplain. There are native speakers of Ukrainian as well as Russian, even though all interviews were carried out in Ukrainian as the veterans’ own language of choice. Despite a lack of participants from parts of Southern and Eastern Ukraine, there are interviewees from far south and east, Crimea and Luhansk, respectively. Others come from Kyiv, large regional capitals, small provincial towns and even villages. There is one participant with PhD and one who only ever graduated from middle school; an actor turned public figure—and a physical labourer from rural area who earns his living from odd jobs. The war experiences described happened on a vast expanse of the frontline from Pisky to Savur-Mohyla, and from Lysychans’k to Volnovakha, but also include Crimea during annexation in one case, and present a balanced panorama of different warzone locations (see Fig. 1).

In-depth interviews were an obvious methodological choice given the scarcity of previous research, which invited qualitative exploration, and difficulty to arrange a participant observation. Moreover, mobile phone use was deemed a neutral issue with a low incentive to conceal facts or distort the narrative consciously. I treat the obtained accounts pragmatically (Kvale and Brinkman 2009, 55), as narratively constructed yet reflective of the experiences; while not a transparent window into war realities, they represent the lived experiences of mobile users as remembered shortly thereafter. While memory is surely selective, the fact that these specific moments connected to mobile communication were remembered testifies to them being “key events” (Moore 2014, 125) and thus emphasized their importance for understanding patterns of mobile phone use on the frontline.

## The Kalashnikov of mobile phones: communication rules of the frontline

The soldier in the modern army under industrial warfare—be it Wehrmacht, Red Army or British Army—was torn away from his kin and social environment, and placed on the frontline where communication with the civilian rear was orderly regulated by the command and censorship through field post. In contrast, the early modern soldier would often take with him his entire family, wife, mistresses, children, who followed in the supply train. The twenty-first century (late modern? postmodern?) soldier is unlike both. He or she is still physically uprooted and removed from their social situation, but the distance is incomparably closer, and the contact never ceases like it was on the frontlines of wars before mobile telephony.

Mobile phone arrives to the frontline in the pockets and backpacks of servicemen and women, as a tangible expression of link to their homes and families—but also to each other. It is the actor that binds the social structure and extends social and familial networks into the war, assimilating war into society. Unlike in the industrial warfare, and the War in Donbas was in 2014–2021 certainly on smaller scale than the industrial wars of the twentieth century, mobiles make the military less isolated from the rest of society and war zone less of a closed area with restricted access. War has become part of peace, and peace has become part of war.

Often these are older phones, already used by the soldier or some relative, or donated by a supportive citizen volunteer. Perhaps it is a brand-new phone from cheaper Chinese and South Korean manufacturers bought at a shining technology store in Kyiv or a regional capital, with an explicit purpose as the soldier’s communication “war horse” that will carry the burden of many information-related tasks. Sometimes it may be bought at a tiny, crammed phone store, a sort of kiosk selling new and used devices, in the midst of a bazaar in the soldier’s hometown or a similar tiny town in the vicinity of the front.

There are two different types of media amalgamations that the mobile phone is built into as soon as it approaches the war zone and catches signal from base stations kilometres away.

The first one is a spatial and synchronous type of media amalgamations whereby different uses are endemic to areas with stronger signal (urbanized and frontline remote areas, where communication becomes intensive, particularly on the web) and weaker signal (rural areas and frontlines, where communication becomes skeletal). In the war zone, mobiles rely on the commercial communication grid created by Ukrainian GSM operators during the 2000s and 2010s, with base stations equipped mostly by Huawei and Ericson, but countryside was deprioritized and has an underdeveloped network (the occupied territories develop their own, Russia-tied infrastructure). All interviewees talk unanimously about the poor connection. The communication grid’s weakness was further aggravated by signal jamming from the Russian side. “Large trucks would arrive concealed as humanitarian aid”, an officer remembers, “and unloaded as little as 10 sacks of sugar while the rest of their load was jamming equipment”.

Another type of media amalgamations is temporal and diachronous. There are clearly great differences between 2014 and 2021 (and some years in between). In 2014, as some interviewees recollect, even walkie talkies were hard to come by, let alone radios, especially for the volunteer units. There was an extreme lack of spare parts for both weapons and communication systems. Some cases of friendly fire are remembered as consequences of the poor communication. A stronger Internet connection, or even Wi-Fi, started appearing around 2016, with the ultimate stabilization of the frontline (even though even in 2014 it might have been available at more stable positions).^1^

**Fig. 2:**
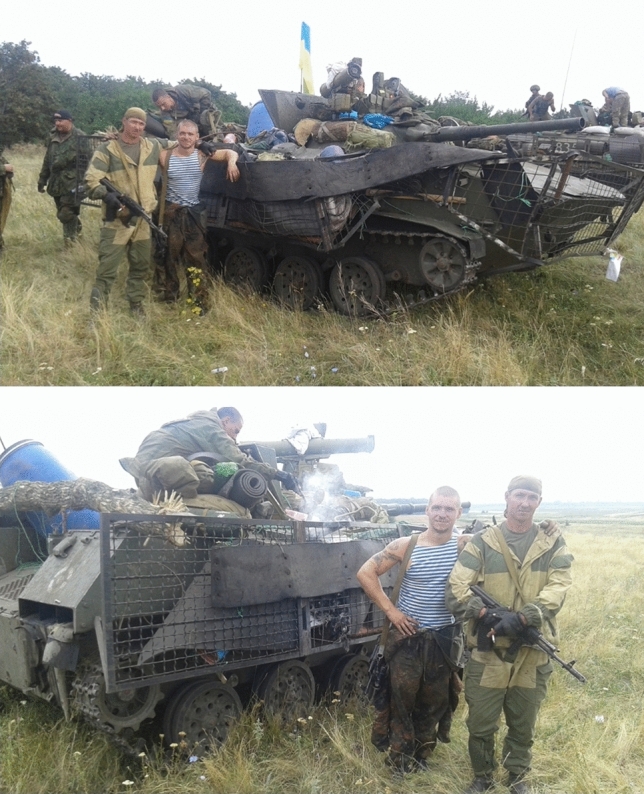
Crews of an IFV damaged in a fresh engagement (courtesy of Valeriy Markus)

One of the first things a late modern soldier does at the frontline is the same as any civilian would do in peace time: reach out for their mobile phone. These compare favourably to other species in frontline media ecology. Laptops are seen as cumbersome and heavy to carry, also a valuable loss. Tablets would break often. Mobile phone, thus, occupies a specific niche shaped by these underlying heterogeneities. While it is seen as generally risky, unsafe and unreliable, mobile is the most convenient and economic way for most combatants to communicate at the frontline and even during combat. At the same time, it is still a marker of hierarchy and class: as important matters tend to be preferably relayed through more secure radio stations used by senior officers (often second-hand and procured from NATO armies or through volunteers), rank-and-file soldiers, like those portrayed by Fig. 2, must rely on cheap smartphones and even on obsolete button mobile phones—a veritable Kalashnikov of military communication. A recurrent solution is to have both at once.

One of the respondents, a pioneer officer, recollects: “I had two numbers and two phones: for home and for service. As soon as I would notice my phone showed up on *their* radars, I would throw it into a river. A cheap one, with buttons. The good one, for home, was kept at the base”. Of course, it is hard to tell whether the phone was really tracked, but it is a testimony of a widespread perception among soldiers; their ways of identifying wiretapping are discussed in a section below.

As a scout soldier noted, the second phone was a salvation in absence of a stable electric feed, when even power banks had already been used up. Simple phones would keep the battery charge around 4–5 days. Cheap, disposable and durable, “the war phone”—dubbed that by one of my respondents—is the most widespread communication tool of this frontline, once again like the war horse of old who “was hardy as his lord, and little cared for bed and board” (to remind of Mazepa’s horse in the poem by Byron).

Smartphones have a more specialized use, because they have a camera and can reproduce multimedia content from the web. At the same time, they are vulnerable to field damage, and the poorer soldiers often find themselves at disadvantage for the lack of a more advanced, camera-equipped and web-connected but more expensive smartphone, like this peasant (a private while in the UAF) from Central Ukraine.

Those who had the possibility, who preserved their phone there, who never had it crushed, yeah those could mess around [povtykat’]. Me, I came with a phone that I crushed on my third day there […]. Well, I just got that kinda stick [dručečok], Nokia, the new one, with buttons. Imperishable. Fall on it, do all sorts of things… The touchscreen, just sit on it once and it cracks, it’s over. I just threw it away, it was no use.

Field phones that operate through wires strung by signal troops form another key element of the media ecology with a different niche. Like cheap button mobiles, they are also economic yet safer means to communicate between established positions. They are used to convey information that really needs to stay confidential for operational reasons because, unlike mobile telephony and Internet messaging, they cannot be hacked into or disrupted without direct physical manipulation. Phone wires are vulnerable to intensive shelling, as some soldiers observed, but this disadvantage is compensated by greater security. The analogue equipment thus reclaims some of its popularity and even prestige, overbearing digital devices. At the same time, this military equipment clearly cannot compete with privately owned mobile phones as means of personal communication, especially with home and family (unimportant to the military’s operational secrecy). Thus, in terms of the overall media ecology, one can observe differentiation and specialization of different communication channels for personal/service uses, while smartphone tends to merge these uses due to its multifunctionality.

Field phones specialize in combat communication at middle level, where top-notch radio stations preferred by higher ranks in staff communication are unavailable. Satellite phones are the most prestigious medium, giving capability to communicate safely even from a concrete underground shelter, but they were expensive and rare, used mostly by high-level commanders. Enlisted personnel admit they avoid it even where available because of the personal financial responsibility for the equipment’s loss or damage.

## Gambiarras of the frontline

Battlefield stimulates improvisation and makeshift workarounds. These contraptions often involved digital technology and civilian hardware converted to military use, sometimes integrated with military hardware in an ingenious way. Soldiers equipped their trenches with electricity (from mobile generators but often siphoned off the public grid), Wi-Fi, freezers, TV sets and even saunas. This is a vivid image of a fortified position as experienced and described by a military chaplain:

Blindages are always connected to electricity, there are lights, some are resting while others are on guard. There is always a laptop and a flat-screen TV connected to CCTV cameras installed around the perimeter so that they would not have to freeze outside in winter cold.

This advanced and comfortable set-up may be characteristic of quieter sectors and second-line fortifications, even though some of the more stable first-line positions came to be equipped like that at later stages. During the earlier, hotter phase in the Donbas War (2014-February 2015), the frontline lacked equipment and stability, and soldiers had other, more vital concerns. One private recollects that Wi-Fi routers only appeared at the end of his term with a special sanction from the company commander.

**Fig. 3:**
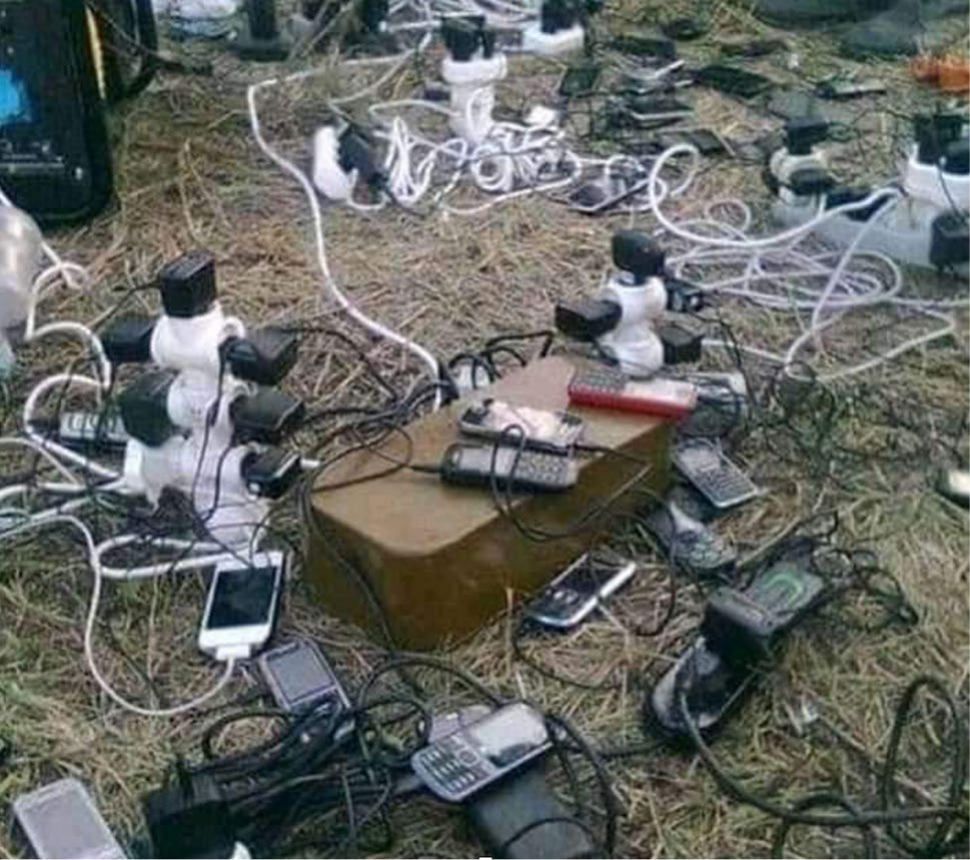
Makeshift mobiles’ charging point, allegedly on the East Ukrainian frontline (courtesy of Facebook)

**Fig. 4:**
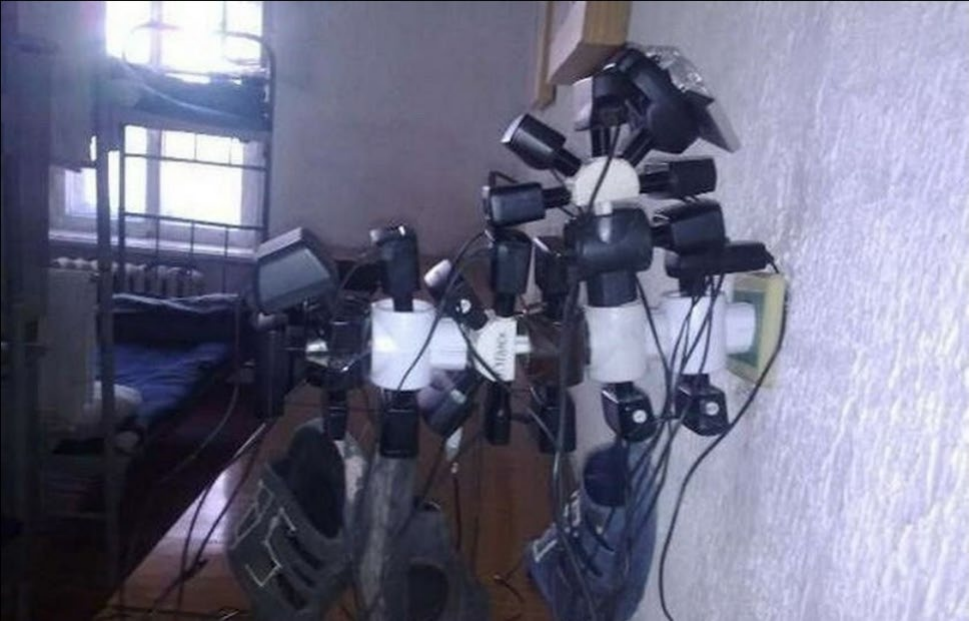
Improvised charging installation at barracks, ca. 2010 (this and all following pictures are courtesy of their author, Valeriy Markus. They date to summer 2014)

The easiest and the most typical makeshift constructed by soldiers in a trench is a charging station for their mobiles. Figure 3 shows one such example—this is an image I found at a military-themed Facebook group but it can be considered verified by one of my interviewees, who instantly laughed at seeing it and exclaimed “but this is how it looked at our unit, are you sure this pic isn’t from us?” Such improvised charging stations are also a well-known staple of the peaceful military life at Soviet-era barracks with very few sockets (cf. Fig. 4).

However, other needs required advanced IT skills. One such area is mapping of own anti-tank minefields by pioneer soldiers using civilian GPS navigators hacked to ensure the acceptable margin of error (1–2 m). Eventually, the need for up-to-date maps brought to life a volunteer-driven project, *Armiya SOS*, whose live, real-time map of the battlefield could be accessed and edited to the minutest detail by soldiers on the ground using standard tablets connected to local network. This project was repeatedly mentioned by several interviewees, apparently fond of its effectiveness.

Civilian professionals also developed a software (“graphic calculation complex”) called *Bronya* (“Armour”) with a purpose to automate all calculations that any canon fire requires (from a light APC automated canon to a heavy 152-mm howitzer). The software is installed on tablets donated by volunteers to the military and is also well known among the respondents. One of them, a gunner, was himself involved in development and introduction of the earliest version in his brigade, which is an interesting example of a participatory practice that influenced decision-making. According to the informant, the software became a key factor in the success of Ukrainian artillery because it allowed to reduce the calculation time from minutes to seconds, which is a light-year difference in today’s warfare. At the same time, the military had to learn from the risks such high-level digitalization entails as one similar app was hacked by the Russians allowing them to target Ukrainian batteries (Martin 2016).

Much of this jury rigging and development of military appliances by civilian volunteers resembles a well-known practice of creating gambiarras, “a contraption that involves a technological recombination of new or given technologies, acting like an intervention in the social sphere in such a way that the contraption itself, often precarious, illegal and transgressive, enters a dialogue with the surrounding community and locality” (Löfgren 2020, 181).

The frontline gambiarras are the material expression of the grassroot character of the war, whereby much of the effort spearheaded and fronted by the state is carried out by a grassroot and almost guerilla work of civilians, junior officers and enlisted personnel. They take participatory war to the level of infrastructure and engineering. All sorts of resources—funds, materiel, expertise and creativity—are invested by soldiers, their relatives and supporters rather than by an assertive and potent state. While this may relate to the special character of the Donbas War, there is still a hint to a self-mobilization potential of Ukrainian society, resembling the total war principles that later came to full implementation in 2022.

There is such a thing in the army as a whip-round for the company [skladatysja na rotu]. When we get salary, the company commander says: ‘Come on, lads, let’s collect 200–300 UAH each, for the company’. […] He collects the money monthly and asks: ‘So, what do we need?’ Say, we move to the front in summer, and we need a freezer because you won’t survive there without it […]. We put aside 5,000 for the freezer. […] Equipment repair is what we spend on the most, everything gets broken all the time, and needs to be repaired at lightning speed. But you can surely raise some 5,000 to lay the internet connection.

Under this situation of a weaker and more passive state, on-the-ground realities of war morph into a more diffused condition, although surely alongside efforts to institutionalize these practices and “arrest” the diffused war within the state framework. Mobile communication hardware, such as smartphones, tablets and GPS navigators, is the actor that binds this military/civilian nexus, which is centred on, enacted through and embodied in technology. While surely not unique (militaries are never fully isolated from the rest of the society), the peculiarity of this nexus is in its participatory character, involvement of small and medium business, NGOs, non-profit civilian initiatives, crowdfunding (often self-crowdfunding by soldiers) and individual creativity. At a time when some researchers found that “civil–military relations appear to be characterized by a widening gap between civil society and governments on the one hand, and the armed forces on the other” (von Bredow 2018, 96), the Ukrainian experience signifies quite the opposite trend.

## Home and war in a pocket

Mobiles maintain soldiers’ ties with home, which often endangers the soldier and his comrades, so the terms for communication must be defined very clearly. Many soldiers describe how they made rules to prevent calls from relatives in the heat of fighting. Often, the rule was that it is the soldier who calls, not the relatives. Ignoring any incoming calls in dangerous situations becomes the norm, an exemption from the culture of connectivity. Many would simply prohibit calling them at any time: “The right time comes, I’ll text myself that all’s good”.

Many, however, do not see “calling the frontline” as something transgressive. One respondent would easily call his comrades on duty when on leave himself: “I simply make a call and see if it is answered. If so, then all is well. If he is out of reach, then he must be in the blindage or under shelling. Then I wait for him to return the call”.

**Fig. 5:**
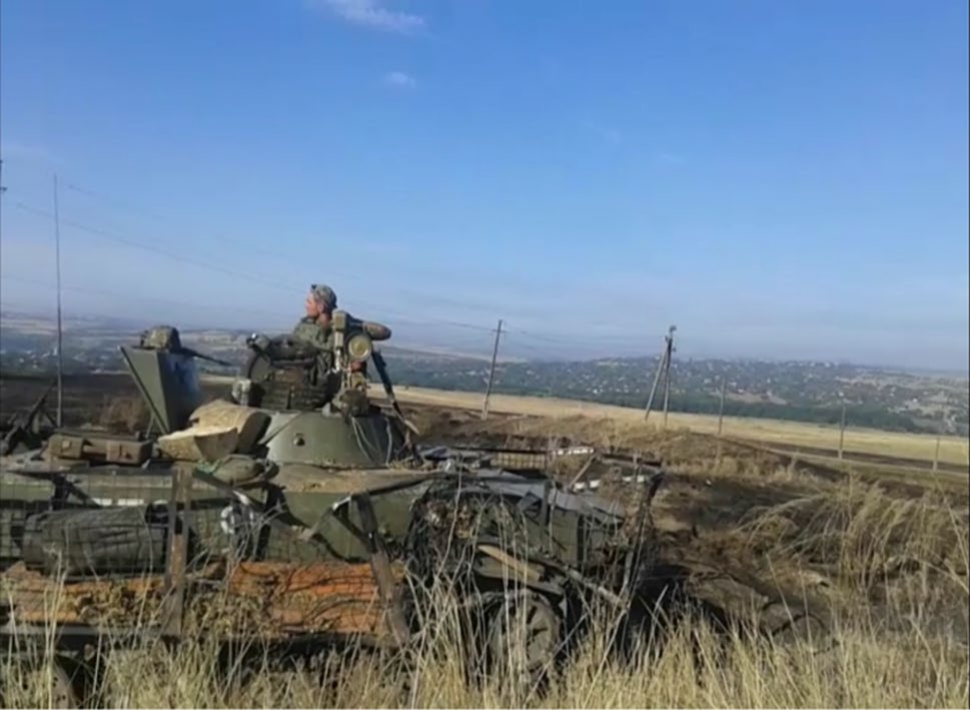
Ukrainian frontline position

In either case, the frontline with a typical position as pictured on Fig. 5 stimulates minimalistic, non-informative and essentially phatic communication whose main content is contact. The main message is the very fact of connection, and the most important information is that the soldier is alive and well. As one relative revealed, “I could instantly figure from his tone whether he could talk. I called with the sole objective, to find out if he was alive”.

Still, the frontline communication policy was often violated by anxious family, and phone conversations occurred even during engagement. “Parents sometimes called from Lviv just as the barrage targeted us. I would hide somewhere and say, ‘I’ll call you back, I’m busy’, so that they wouldn’t hear that cannonading”.

These dangers, normally remote and imagined but now brought home in vivid mediated images (sounds of shelling in real time, photographs, videos), are clearly unsettling for families. Simultaneously, soldiers also feel pressed to maintain emotional stability of the close ones. As a result, well-intended deceptions become more normalized than in peaceful everyday life.

Some avoided telling outright their loved ones the truth that they were now at the frontline and not at a training unit. “We ain’t pick the phone. Cause just as you pick, they go like, ‘Oh sweet Lord, what are you up to poor things?’ The best is not to tell you’re in the thick of it. You hide deep and tell you’re at a base”, an enlisted private from a rural area mused. While many, including the spouses I spoke to, believe that the permanent connection made life easier and even reduced anxiety and stress, another respondent, a junior officer, reflected on how he imagines the feelings of his mother and wife. “I think it was actually harder for them because of the possibility to call. It was better for them not to know where exactly I was and what was happening to me”. Some of the respondents noted that the family had quickly figured these “little lies”, and the entire communication turned into a game where both parties pretended to believe one another.

There are also those who could use their situation to manipulate relatives emotionally. Thus, the context of relationships back home extended to the frontline and integrated it:

“We had a hutsul guy from Transcarpathia who called his wife at night and said: ‘Oh, Halya, there’s real hell over here…’ A single mortar shell landed like hundreds of meters away, and he goes like ‘this is hell, I ain’t know if I live to see the morning!’ Just to raise his authority, make himself a victim or a hero. There are many like him”.

**Fig. 6:**
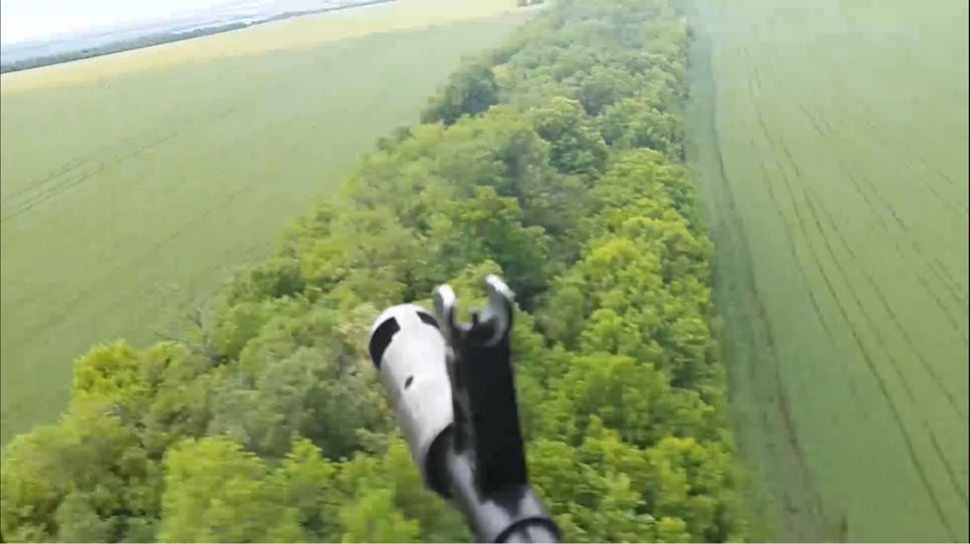
Donbas warzone filmed from a UAF helicopter

Temporal change is substantial, too. The combatants in 2014 felt that they were navigating an unknown territory where many things were possible and felt the urge to share their new experiences (Fig. 6). “At first it was like ‘hey mom, look at me’. That was a real case. I saw a guy who was (lightly) wounded and showed it to his mom via a videocall: ‘Mom, look I am wounded’ as he bled”. Later, with war becoming static and routine, and after many hard-won lessons, this attitude was replaced by complex rules and avoidance of unnecessary risks. One could observe a gradual shift from that “call mama” mentality to communicating from safer bases behind the frontline, in pace with professionalization and routinization of the conflict.

War also raises the status of voice and oral communication, seen as more authentic and trustworthy than suspicious text. Voice calls were in particular prioritized over texting. The soldiers stress that it was vital to hear the voices of their wives and especially children, and spouses describe the effect they experienced as therapeutic as well. Texting, on the contrary, is pictured as fishy: “When you get a text, God knows who is actually texting. When you text, God knows who will read it on the way”. This leads to another broad area of frontline media ecology: wiretapping.

## “God knows who is listening”

The mobile phone is among the soldier’s first non-human allies alongside their weapon, but it is not a trusted one. If it is still a friend, it is the kind of friend you would not let in on the most important secrets. In such extreme conditions the “basic trust in justness and non-failure of these systems” (Jansson 2018, 73) that legitimates mediatization is extremely low. Electronic eavesdropping pervaded the frontline of the War in Donbas, and the relevant equipment was used by both the Ukrainian and separatist/Russian units. Walkie-talkies, mobile phones and even radio stations were monitored, and commanding officers tended to discuss issues that matter in person whenever possible. Surveillance is not an abstract concept on the frontline—it is the lived reality.

**Fig. 7:**
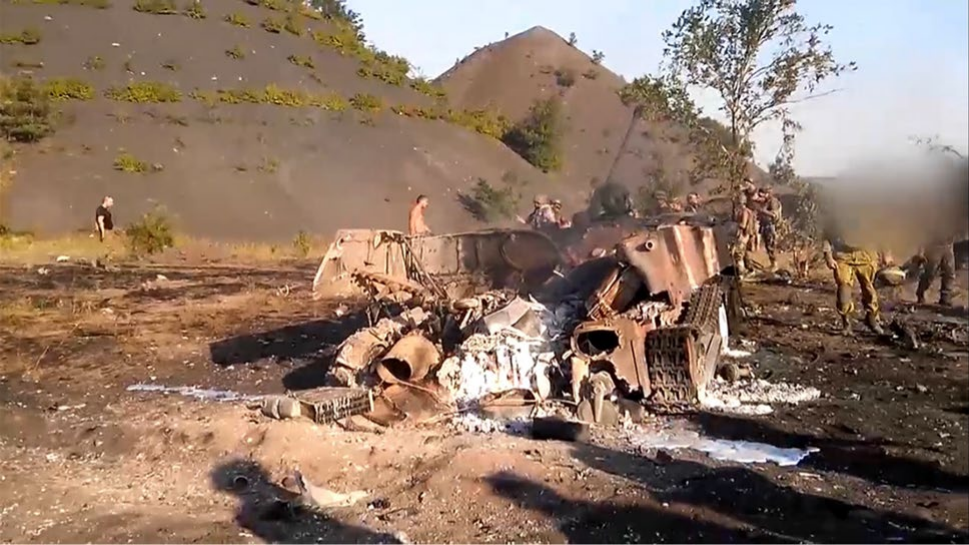
Consequences of shelling

**Fig. 8:**
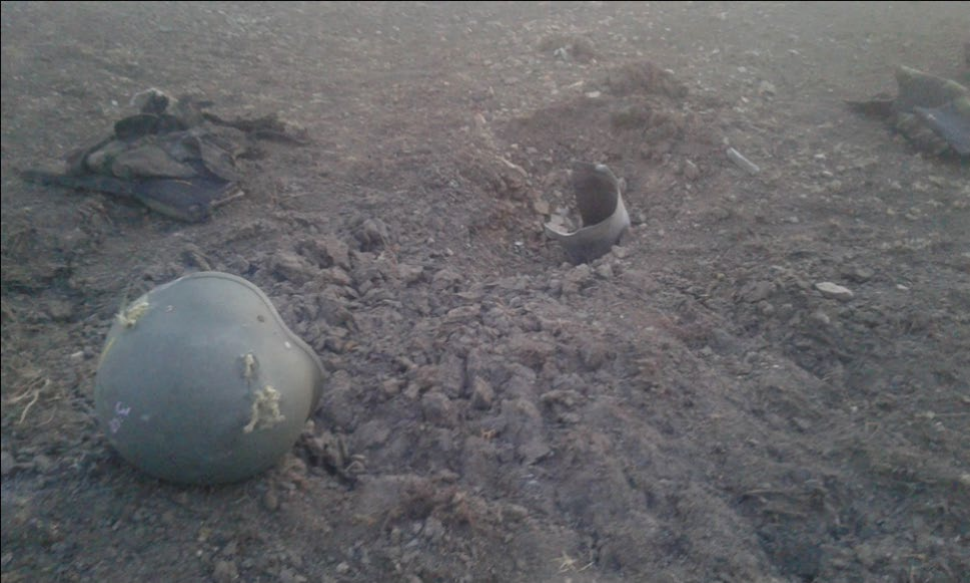
Consequences of shelling: debris and damaged gear of the killed

This communication literacy to the point of distrust also developed from trust that initially was excessive. A pioneer officer recollected how he was instructed by his senior commander in 2014 to report on a task by sending a picture of the installation in question to a private email on the Russian service Mail.ru, which was almost as good as sending it directly to FSB. Such practices no longer happen, as the informants testify (Figs. 6, 7 and 8).

Wiretapping, though, remains a problem and is aggravated by the similar equipment used by both sides. The cheapest and most hack-prone appliances are also the most widespread: “Just turn on the autosearch, and it finds by itself all the nearest active signals. […] you listen to it and all”. But Ukrainian soldiers did not feel they were merely passive victims, and freely admit they often did the same.

Back then we used […] old Motorolas with a real mighty antenna, both from volunteers and organic equipment [štatni]. We mostly used the Chinese junk, and we could listen to them via that autosearch function. Our guys would come once a week to reflash it [pereprošyt’] so that they couldn’t do the same to us.

Soldiers were also used to permanent wiretapping not only by the enemy (rumoured by some to reach up to 25 km deep behind the Ukrainian lines) but also by what they perceive as eavesdropping by their own security services, something that my respondents believe in unanimously. The conversations are made banal on purpose, and certain keywords are avoided altogether: “Once my wife called and asked, ‘where are you now?’ And I heard at once a characteristic sound in the background, meaning they are recording. I say, ‘Guys, turn it off, let me talk with my wife, I’m legit’”.

Many obviously found relief and a touch of empowerment in talking to the supposed wiretappers and ridiculing them. “It was audible. Either an echo or clicking, or else some rustling. I go: ‘Wassup guys, how ‘bout some war today?’” The inventory of what is popularly believed to be a sign of wiretapping includes voices that interfere with phone conversations, or even stranger things:

At the tip I had H + most of the time. Sometimes it disappeared and a 4G would appear instead. Then we were told to turn off our phones, and experienced lads who had stayed there long said it was 99% certain that the phone was being hacked into by the enemy as it switched to the signal from their cell site, not ours.

Although many, particularly the signal troops serviceman I talked to, doubt the possibility to identify hacking by noises and network jumps while the device catches signal from a new station, this does nothing to the fact that such stories are a core element of the soldier lore, remain widely believed and provoke counteractions, such as code words as a strategy to resist eavesdropping: “For example, I can call and say, ‘the table is served, waiting for guests.’ Then, ‘guests on the way, serve hot meal’, and there goes the artillery”. Other soldiers use numerical codes; for instance, 450 can stand for “all clear”. Real or not, this perceived wiretapping is part of soldier’s world and mobile use.

On the other hand, soldiers admit their phones would catch signal from base stations from “separatist”-run and Russian mobile operators in certain areas, which obviously creates opportunities for tampering with their data. The informants cite documented cases of Russians installing a fake base station masked as that of a Ukrainian operator, with a purpose to get access to communications through the mobiles of soldiers deployed in the area. Russian intelligence may also target soldiers with online gaming addiction by identifying devices with large traffic to gaming sites, to use this addiction in their recruiting efforts. For all these reasons, online communication has been restricted. While Skype, Telegram (until about 2018) and WhatsApp were at least allowed, the use of Viber was banned (due to its poor security and Russian links) but it still may be used for planting disinformation with the enemy. Others, though, continue using Viber but avoid going into any significant and potentially sensitive details. “We were instructed to disinform via the phone, even the battalion commander told us: ‘You talk to parents, friends—mess your traces [putajte slidy]’”. According to others still, the connection was often too poor even to call, let alone use online messengers. Short-range wireless and peer-to-peer tools, such as Bluetooth and SHAREit, were seen as safer alternatives to send files to comrades-in-arms.

And yet it was this communicational no man’s land, where enemy infrastructures intertwined and enmeshed, that created a chance for contact, and a chance to subvert eavesdropping by explicitly addressing those who eavesdrop. Mobile and cheap walkies were the actors that make such encounters possible.

We bought two [local] SIM cards because some operators had no coverage in the area. We started at once getting SMS urging to defect to ‘DPR’. […] We tried calling them just for fun, to chat and ask ‘so, how can we enlist’—but nobody answered […]. Sometimes in Pisky we contacted the separs via walkies and swore at each other. Well, that too was showing off [ponty] because smart people would listen to what the enemy is talking about rather than argue.

And there were obviously such smart people who would even occasionally find enemy phones with SIM cards and use them:

Many phones get dropped and a sabotage-and-reconnaissance group can pick one believing it is ours. Until they figured who they are talking to, they would talk to us for a time, and we could ask and get useful information. One such phone would lie at ours for a month, and calls were coming all that time.

But even in showing off, soldiers enjoyed the feeling of power and gaining control from openly announcing their imminent attack: “Of course we talked to them. Before our salvo, we would drop to their walkies: ‘Get cover guys, here we go’. That was some fuss [kipiš] there: ‘Who said that?’ [the interviewee quotes in Russian, laughing]”. An old acquaintance from “the other side” could sometimes call to warn about the forthcoming attack, an event described in the fictionalized war memoir by Valeriy Markus (Ananyev 2019, 245–249). Thus, communication devices—walkies but often mobile phones—become mediators that associate not only friendly actors but also belligerents themselves.

## Soft targets: targeting and spotting

The phone can let you down in many ways. Even as the phone’s screen lights up in darkness, it may be visible miles away and become as dangerous as lighting up a cigarette. Even phone directories can be risky.

Our company commander had a notebook with our contacts and necessary contacts to next of kin in case of trouble. There was one critical moment when the risk of massive shelling was imminent, and we had no time to pull away. I saw the commander simply destroy the notebook. He made a small fire and burned it.

Another danger the phone may bring on its owner is fire by enemy artillery (Figs. 7, 8). Here is one of the shocking cases from an experienced officer:

I went with a guy who had to broadcast coordinates. I cleared the field, he set it up, then I check with the map and see we came to a wrong area. He says,’I know.’ And then the area where we were supposed to be is shelled. He says, ‘Got it?’. I say,’Got it.’ And then a new strike hits very close to us! And the guy goes nuts:’What bastard took the phone? I’ll kill ya!’ And a young soldier comes forward and says, ‘I just called mama.’ In 5 min we were gone, and the place was pulverized.

A more routine situation is when artillery targets many mobile numbers that simultaneously become active in an unexpected place, such as in the middle of a field or wilderness. Tracing this requires access to base stations, which is often unproblematic at the digitally porous frontline. One informant told me it is even possible to calculate the unit’s strength by weighing the number of active phones with a certain factor (the expected number of people who routinely turn off or leave their phones behind—apparently the military tried to gauge it).

Aware of the risk, soldiers are also trying to use it to their advantage and target the enemy phones. “When our mortars started shelling, they asked us to turn off our phones that interfered with their targeting in some way. As soon as they were done, they let us turn them back on”. However, as the gunner informant admitted, this job is often done by highly specialized units, is not advertised and somewhat exceptional. At the same time, some reports indicate that specialized devices, privately owned by some of the troops, could be used to identify mobile phone signal.

Part of the explanation is that there were too many mobiles anyway, which created much noise and decreased the relevance of targeting mobile phone signals. Introducing the human actor raised the network’s precision. Spotting was also done by mobile and often involved civilians (a further blurring of the military/civilian line). By 2018, most of the spotters would have been caught, but they had been an ordinary element in the fighting earlier.

Like, a guy with a phone, who is riding on a motorbike and shouting, ‘to the right’, ‘to the left’. We see someone like that, we send out an assault team. There is even a base of the numbers those spotters call. We didn’t have those and had to correct with a drone.

The strategies employed to deal with the targeting risk are a lesson in the futility of prohibition. Commanders tell to leave phones at the base often yet inconsistently because it is inapplicable for some tasks and there would always be those who disobey. In July 2015 the Ukrainian parliament banned the use of mobile phones, cameras, radio receivers and computers by soldiers in the warzone. Mobiles remained in use de facto as the most reliable communication tool in absence of secure systems. The effort refocused from banning what cannot possibly be banned to coping strategies. In particular, activists published soldier’s guides on the use of mobiles, with advice ranging from “no earplugs!” in the field to how to protect a smartphone from mice (Teksty 2015). Eventually, mobiles were allowed again in August 2017 after an outcry and what soldiers described as mass disobedience of the personnel. Mobile use may now be officially permitted by respective commanders if necessary for performing a military task, but soldiers point out that most of the ways in which they use phones can be excused as connected to a military task. Communication is vital. It is the air the war breathes.

In other words, people had to enter a complex negotiation of the frontline connectivity consequences. The disadvantages of turning off the phone are self-evident, such as being unable to communicate during combat, but sometimes appear too basic. One soldier complained that, as their unit was told to turn off their phones and dislocate batteries amid incessant enemy shelling, he had a difficulty with his sentry duties. “I just couldn’t follow the time because I had no wristwatch. I just simply couldn’t tell when my duty was over, and when it was my turn to do sentry. Imagine not sleeping a few nights before, and that we were only two to cover sentry all night”.

Phone is thus often seen as an acceptable risk: people are not very concerned with security. Liberty trumps safety, as often in Eastern Europe, especially when safety appears as illegitimate and whimsical imposition in absence of ostensible threat in a place known to be dangerous anyway. To be sure, civilians are reported by my respondents to be as much or even more negligible. In any case, there are many other ways to find information and target fire, so abstaining from the phone use does not remove dangers while taking away all the benefits, and it makes little difference to many whether one dies in a shelling targeted through one’s own phone or an enemy drone.

Safety was also less significant under conditions of stagnant, static warfare. “Everyone knows anyway that such position exists”, a military paramedic explained. She continues:

Fighters are aware [of the risks] but it is perceived differently there. When you’re in it for years, people stop caring for [zabyvajut’ na] armour vests, helmets, because you can’t escape your fate. […] If you’re having a smoke under barrage, so [why care about the phone?]. I saw people using mobiles under shelling. […] Myself, I didn’t turn it off when we were under artillery fire. Conversely, I felt an urge to send farewell texts to all who I know. Really. But I didn’t do it because… what if not, what if I survive? [laughing].

## “I watched all seasons of Game of Thrones there”

One of the most vital smartphone functions, and our major dependencies on them, is providing access to news content. With official military media unpopular and dismissed as “brainwashing”, Facebook—accessed overwhelmingly through smartphones—became the principal source of news (on par with relatives’ word of mouth). This forced soldiers to sort out and fact check a variety of sources by themselves and to develop media literacy skills. Some are now aware of fake news and manipulation, and many developed that awareness first after they had arrived on the frontline.

Under these conditions, it was again the power of oral word that held the most authority. As one officer remembers, “To get the news, we called each other”. However, in words of another interviewee, a journalist in civilian life, “soldiers are not very interested in information about the war”. Actually, the content in high demand is entertainment.

Mobiles, and to a lesser extent tablets and laptops (especially at the “second-line” bases), provided entertainment on what interviewees oftentimes describe as a “boring frontline”. “It is *very* boring. You get used to that circus [balahan], and then it gets so silent and you think: ‘There’s something fishy about it’. And weird thoughts start coming to your mind”.

**Fig. 9:**
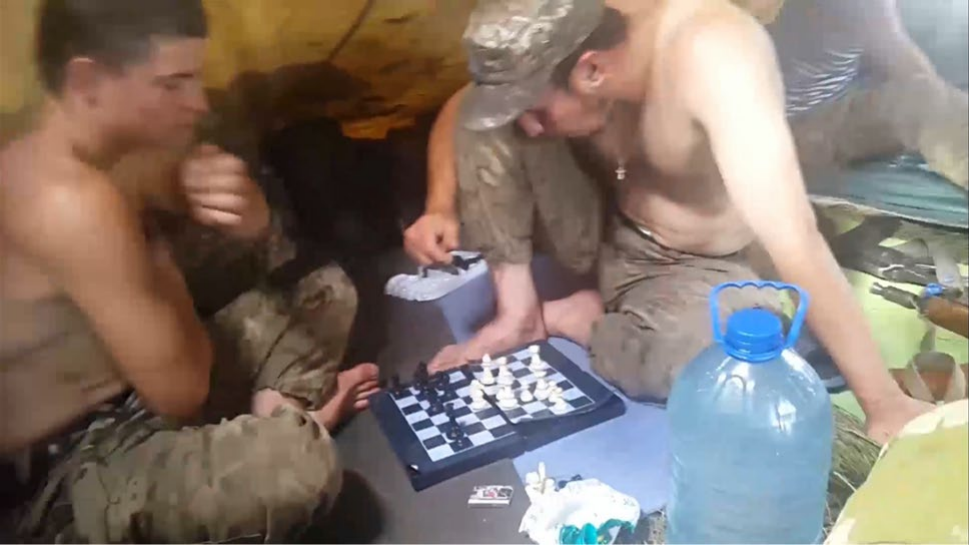
Pastime at the “boring frontline”

Soldiers used phones to watch films and series, play games (such as mobile versions of chess that could also be played as a boardgame, pictured in Fig. 9), listen to music, watch TV and receive news on the frontline. This was also stimulated by the systematic TV signal jamming from the Russian side (reportedly up to 15 km deep). Poor connection stimulated use of torrents that allow downloading about one hour worth of content a day even with unstable connection.

"We once occupied a position for a month during Christmas and New Year holidays. It was a ceasefire […]. We were underground, the weather was a nightmare, snow and rain for a month in that Donetsk clayish soil. Knee-deep in mud, and no internet. We ran around: ‘Who’s got movies?’ There, I watched every but the last season of *Game of Thrones*—on the phone. The guy copied to my 64 GB phone memory card as much as could fit, I watched and deleted it to make room for the next batch".

Information sharing would thus become a communal act: “Those who hanged out at the headquarters, in more civilized conditions, would simply download stuff to a memory stick and pass it to us”. Also, media amalgamations would form around places with better infrastructure and stronger bandwidth. “We often used the fact that Bakhmut had a better 4G connection. It was our downloading hub”.

Many soldiers read books, and mostly on mobiles or tablets with saved ebooks, effectively getting rid of the extra weight and increasing the amount of available reading. The priest, who since 2015 is dividing his time between his parish in northern Ukraine and the frontline units he services, says that he never leaves for the warzone without his smartphone, tablet and laptop.

The weight of all the service books I need to carry would have been prohibitive, but I have all of them electronically on my tablet, as well as music and records of liturgies. I am carrying out different rites, including marriages and baptism, because I am also trying to embrace local civilians with spiritual care to the best of my capacity as I have founded a church and a parish in a frontline village.

**Fig. 10:**
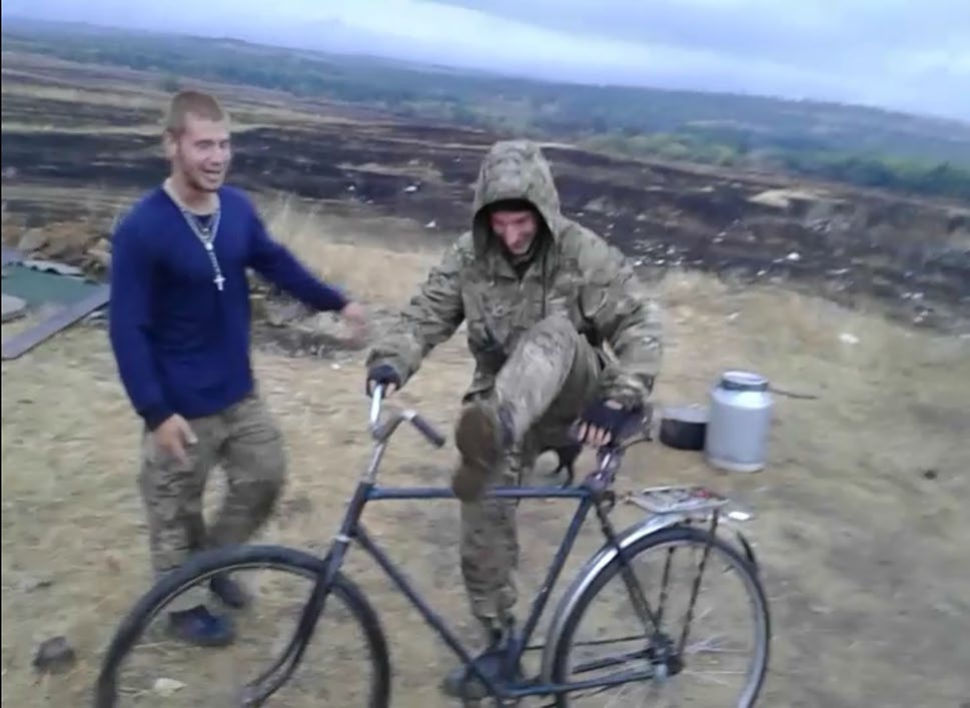
Still from a mobile video prank at frontline

**Fig. 11:**
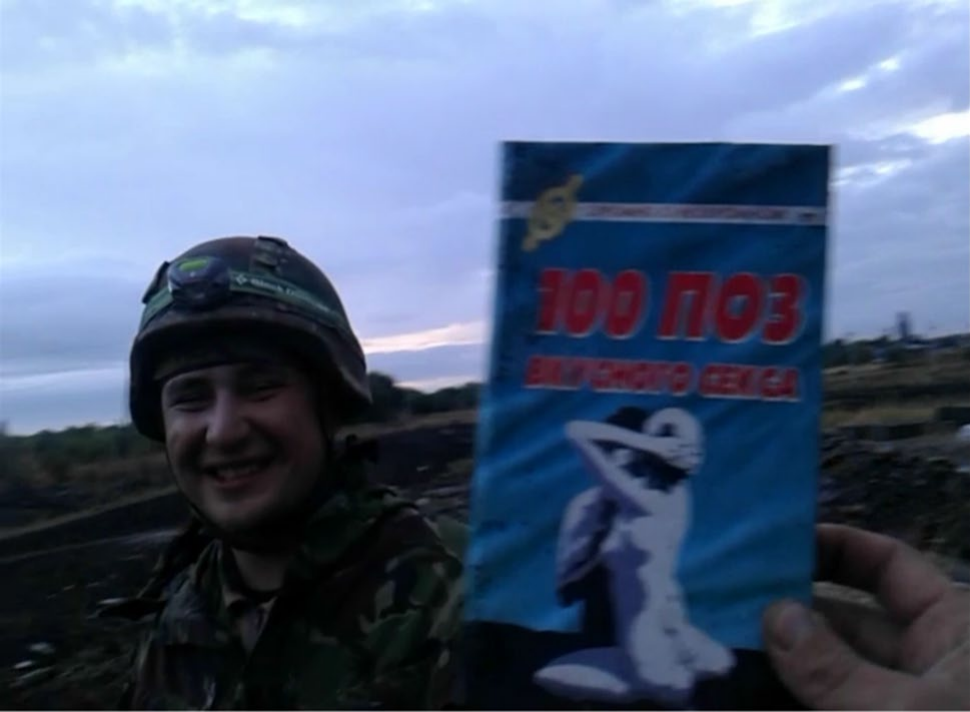
Still from a mobile video prank at frontline (the title of the book found in an abandoned enemy trench reads "100 Positions for Tasty Sex")

Another important affordance of the mobile is the presence of camera. Taking photographs and filming was part and parcel of killing time on the boring frontline. These participatory practices could be as simple as making video pranks, exemplified in Figs. 10 and 11, or in this story from a private:

It was prohibited but who would tell you anything if nobody ever visited us? All fear for their butts [očkujut’]. […] I even have a memory stick full of all those pranks. Like, we would be bored to death in winter, and would take a hood from a *Žygul’* car, wire it to another car and ride on it like a sledge through the town, with pirouettes [vydzegeljuvaly].

Many saw video shooting as entertainment and created own archives of mediated memories. Given that for some of the early combatants it was an adventure, a form of military tourism, such attitude is hardly surprising. On the other hand, cameras had a practical role as a tool to document events, such as POW interrogations or enemy shelling (especially in the context of OSCE monitoring mission tasked with registering all skirmishes and shelling as part of the organization’s peacemaking effort). The omnipresence of recording also enabled the rise of unusual “war porn” genres, such as filmed phone conversations with the relatives of soldiers killed in action (practised by Russian irregulars) and the spread of frontline blogging, encapsulated in the figure of Valeriy Markus (Ananyev) and his rise to a practically celebrity status.

## Conclusion: between “mobile lives”, “immobile lives” and “mobile deaths”

What are the complex and contradictory ways in which mobile phones become integrated in warfare? Firstly, mobiles are omnipresent despite formal bans. This created a produsage environment characterized by 24/7 connectivity and a digitally porous frontline (as devices routinely catch signal from enemy territory). Through it, a specific frontline participatory culture arose that encouraged innovation, technological improvisation and creativity in trading verbal abuse with the foe or staging video pranks. It is often based on rational choices and demonstrated potential for subverting top-down regulations and bans through disobedience and practising the prohibited mobile communication. Disobedience influenced decision-making as the failed bans, which most of the soldiers ignored, were eventually scrapped. This demonstrates that participation can have a political dimension (Carpentier 2016) even in strictly regulated environment of an army at war.

Some aspects of this media ecology are akin to civilian experiences. However, unlike in Jansson’s analysis focused on middle class and on “mobile lives” as a token of prestige, the soldierly lives—largely immobilized by the stagnant frontlines of a frozen conflict—demonstrate how mediatization becomes a part of the low-class experience, where not only death itself is not spared from mediated produsage, but the media turn out to be a matter of life and death, becoming intertwined with killing and dying.

Mobiles are used for wiretapping, fire targeting, minefield mapping. A variety of personal purposes, such as intimate communication and entertainment, are combined in the same device with combat communication that supplants old or unavailable equipment and fills gaps in military infrastructure. At the same time, the mobile increases risks to be wiretapped or targeted. Thus, it manages a complicated regime of access: opening the military to civilians, on the one hand, and to the enemy, on the other. This makes it a mediated extension of battlefield, pulling new actors therein and forming new associations between all of them.

The broad use of mobiles in the warzone disrupts the closed nature of the military as state institution and “an island within society-at-large” (Soeters et al 2014, 3), introducing new corporate actors beyond the traditional weapon manufacturers to the frontline concatenation, now embracing mobile phone producers, Big Tech, small local IT enterprises. As a non-human actor, mobile is a focal point that bundles participatory efforts of civilian individuals, volunteer groups and small businesses to support the army. Mobile is the hardware on which the participatory war runs. Of course, smartphone use involves also social media companies and their culture of connectivity, dataveillance and algorithmically coded sociality (prone to manipulation by third parties) as a factor and a new actor in war. Accordingly, the boundaries collapse between the state (the military), the corporate, the private and even the intimate, creating a unique interconnected re-figuration of social situations and networks around war. War communication becomes part of the profit-generating mobile telephony and social media business, and that business in its turn supplies the military with a combat tool, which a soldier can use both to kill the enemy and to talk to his or her kids minutes later.

Consequently, mobile phone is both an asset and a liability in combat that requires soldiers to work out clear but informal rules around its use, and to integrate the logics of both connectedness and disconnection, of mediatization and counter-mediatization. The mobile phone has actively intervened in the course of war in Donbas and functioned as a mediator in the human associations necessitated by war. It can provide just enough edge over the enemy in combat. But it is also the actor that helps stabilize, support and make resilient both the army as an institution, its link to the civilian support base that consists of relatives and volunteers, and each individual member’s network of social ties. It extends the lifeline from the civilians/civil society and their networked publics (cf. Boichak 2021) to the army, often bypassing all other state institutions, and in so doing enables the crowdsourced and participatory war. It makes the nexus of the Ukrainian army and society more robust in confrontation with more powerful but state-centric Russian military machine, and it represents a communicative refiguration of the military.

Moreover, the modern soldier is no longer possible without a mobile (something that is also confirmed in the context of a large scale interstate war raging in Ukraine since 24 February 2022). It is no longer a mere means to keep in touch and have fun, wisely left at the base before the expected engagement. It is an inherent, integral part of the assemblage of engagement and war—this was true for the hybrid Donbas War in 2014–2021 and remains true for the 2022 Russian invasion of Ukraine, the latest stage of the Russo-Ukrainian War. This war is fought in large part with a civilian tool, a corporate product, a private belonging and even an intimate object turned into a weapon, an ultimate form of hybridity. Inasmuch as Clausewitz’s war is a continuation of politics by another means, and politics itself now, in the era of hybrid warfare, is a continuation of war with other means, the war phone is also a political object. That also points to the future directions. How does this alter the very concept of weapon? And how does the digital war waged from a pocket and a palm of hand release the arrested war from its arrest?

## Note

1. For example, Valeriy Markus describes a commanding post at a coal mine as enjoying Wi-Fi in August 2014 (Ananyev 2019).
